# RBUD: A New Functional Potential Analysis Approach for Whole Microbial Genome Shotgun Sequencing

**DOI:** 10.3390/microorganisms8101563

**Published:** 2020-10-10

**Authors:** Zhikai Xing, Yunting Zhang, Meng Li, Chongye Guo, Shuangli Mi

**Affiliations:** 1 Key Laboratory of Genomic and Precision Medicine, Beijing Institute of Genomics, Chinese Academy of Sciences, China National Center for Bioinformation, Beijing 100101, China; xingzhk@big.ac.cn (Z.X.); zhangyunting17m@big.ac.cn (Y.Z.); limeng@big.ac.cn (M.L.); 2 University of Chinese Academy of Sciences, Beijing 100049, China

**Keywords:** microbial community, metagenome database, metagenomic profiling, microbial function, type 2 diabetes mellitus (T2D), avian colibacillosis

## Abstract

Whole metagenome shotgun sequencing is a powerful approach to detect the functional potential of microbial communities. Currently, the read-based metagenomics profiling for established database (RBED) method is one of the two kinds of conventional methods for species and functional annotations. However, the databases, which are established based on test samples or specific reference genomes or protein sequences, limit the coverage of global microbial diversity. The other assembly-based metagenomics profiling for unestablished database (ABUD) method has a low utilization rate of reads, resulting in a lot of biological information loss. In this study, we proposed a new method, read-based metagenomics profiling for unestablished database (RBUD), based on Metagenome Database of Global Microorganisms (MDGM), to solve the above problems. To evaluate the accuracy and effectiveness of our method, the intestinal bacterial composition and function analyses were performed in both avian colibacillosis chicken cases and type 2 diabetes mellitus patients. Comparing to the existing methods, RBUD is superior in detecting proteins, percentage of reads mapping and ontological similarity of intestinal microbes. The results of RBUD are in better agreement with the classical functional studies on these two diseases. RBUD also has the advantages of fast analysis speed and is not limited by the sample size.

## 1. Introduction

In recent years, with the improvement of high-throughput sequencing technology and the rapid development of microbial research methods, it has been possible to systematically analyze all microorganisms in samples, not just those that are amenable to cultivation. Previously, these methods were mainly applied to taxonomic studies of microorganism using phylogenetic information genes (such as ribosomal RNA) [1,2]. Moreover, these studies provide a new perspective for us to understand the essential role of microorganisms in human health, soil ecology, environmental remediation and many other fields [3,4,5]. However, due to the similarity of rRNA sequences and different functions of microorganisms in different environments, it is difficult to expand the understanding of their functions through taxonomic research [6].

Whole metagenome shotgun (WMS) sequencing data can guide researchers to focus on the whole microorganisms as a community, and classify the internal genes and protein coding functions by assembling these data into an annotated reference database [7]. The main processes of the current approaches are sequence alignment, assembly and subsequent annotation, which have high requirements for mapping rate, large sample size and computer resources [1,7]. At present, there are two kinds of prevailing strategies for the analysis of whole genome shotgun sequencing data, including assembly-based profiling and read-based profiling [8]. However, these approaches are greatly restricted. The former requires reads splicing, contigs assembly and prediction of open reading frame (ORF) before mapping the data to the reference database [9]. In all these steps, data utilization is reduced due to the loss of low coverage areas [8]. The latter requires a reference database of specific host species, such as Integrated Microbial Genomes and Microbiomes (IMG) and Metagenomics of the Human Intestinal Tract (MetaHIT) for humans in order to perform conventional analysis procedures [10,11]. There are also a small number of databases for other host species in metagenomics studies, including the chicken [12], pig [13] and mouse [14]. However, the construction of these databases requires a large number of sequencing data and sample sets, and the cost is very high [8]. This makes it difficult to succeed in the studies for small samples and uncommon host species. Therefore, the current methods are not sufficient to achieve the functional metagenomics studies for the lack of existing metagenomic databases and small sample size. Moreover, these methods cannot effectively improve the utilization of sequencing data.

In this study, we developed a new method named read-based metagenomics profiling for unestablished database (RBUD) for metagenomics studies. In order to improve the utilization of sequencing data, the RBUD method was optimized by omitting the steps of contigs assembly and ORF prediction. Therefore, the RBUD method could shorten the time of data analysis. More importantly, since RBUD contained an establishment step of a database pertaining to microorganisms with different resources to expand its application, it is a great assist for small-sample research, which can avoid the lack of a reference database. In the actual cases, our analysis by RBUD successfully identified target genes and the pathogenesis of pathogenic bacteria. After running, our method can generate a report document containing comprehensive analysis results, including species abundance, gene abundance, gene ontology, pathways and antibiotic resistance.

## 2. Materials and Methods

### 2.1. Study Inclusion and Data Acquisition

#### 2.1.1. Animals

This study was approved by the ethics committee of Beijing Institute of Genomics, Chinese Academy of Sciences, China National Center for Bioinformation (No.2014S010, April 2014). Bacterial DNA were extracted from the cecum contents of 18 broiler chickens (Rose 308), including 9 healthy controls and 9 avian colibacillosis cases. All chickens were raised on standard farms in Jiangsu Province, China. The samples were collected in the aseptic laboratory (Table S1). The DNA were sequenced and published as CRA000950 according to the records of the Genome Sequence Archive (GSA) of National Genomics Data Center (Table S2). The use of antibiotics is in accordance with the provisions of the Administration of Feeds and Feed Additives (2016) issued by the Ministry of Agriculture of China.

#### 2.1.2. Human

The published fecal shotgun metagenomic data of type 2 diabetes mellitus (T2D) and healthy controls were searched through PubMed. The patients were diagnosed with diabetes at the time of initial diagnosis according to the definition of World Health Organization (Table S3) [15]. Sequencing information of the samples was retrieved from previously published data (Table S4). Raw FASTQ files were downloaded from the Sequence Read Archive (SRA) of the National Center for Biotechnology Information (NCBI), using the following SRA identifiers: SRA045646 and SRA050230, consisting clinical data of 368 individuals and shotgun metagenomic sequencing data of fecal samples [16,17]. In order to construct small sample data for further analysis, we first screened out 104 healthy individuals and 94 T2D patients with all physiological indicators, including body mass index, fasting blood glucose, systolic blood pressure, diastolic blood pressure, fasting serum insulin, fasting serum C-peptide, glycosylated hemoglobin triglyceride, total cholesterol, high density lipoprotein and low density lipoprotein. Then, 10 people were randomly selected from each group as sub-sequent research samples.

### 2.2. Raw Data Quality Control

The chicken (ftp://ftp.ncbi.nlm.nih.gov/genomes/Gallusgallus/; galGal4) and human (ftp://ftp.ncbi.nlm.nih.gov/genomes/Hsapiens/; GRCh38) reference genomes were downloaded from the NCBI database. Reads from different host sources aligned with the corresponding host genome (alignment with SOAPaligner/soap2, version 2.21) were deleted [18]. After that, they were trimmed if their quality threshold were equal to or less than 20. Any reads less than 25 bp were removed in the read pairs sequencing using SolexaQA software (version 2.2) [19]. The remaining reads were considered to be high-quality reads for further analysis.

### 2.3. Illumina Hiseq 2500 Short Reads De Novo Assembly, Gene Prediction and Construction of the Non-Redundant Gene Set

High-quality short reads for each DNA sample were assembled by the SOAPdenovo assembler, and the parameters “-K 23” were used to indicate the minimum sequence overlap required [20]. MetaGeneMark software (version 3.25, Atlanta, GA, USA) [21,22] was used to predict a series of ORFs based on anonymous genomic sequences to identify ORF from the contigs of each sample, then the ORFs with minimum length of 500 bp were selected for further study [23,24]. Using Cd-hit (version 4.6.1, San Diego, CA, USA), the predicted protein coding genes with minimum length of 100 bp were clustered with 95% sequence consistency, and the parameters were -c 0.95, -n 8, -s 0.9, -M 2500 [25]. The predicted genes were aligned against the annotated reference databases by BLASTN and BLASTP (e-value < 1 × 10^−5^) to identified species and functional annotations [26]. Finally, a non-redundant gene set was constructed for future analysis.

### 2.4. The Basic Process of Establishing Taxonomic Abundance Profile and Functional Abundance Profile

The high-quality reads of each sample were aligned with the predicted genes mentioned above or the existing gene catalog by SOAPalign software with parameters of “-r 2 -m 200 -x 1000”, respectively [18]. Then, the sequence-based abundance profiling was performed as previously described [27].

To construct the taxonomic abundance profile, we used the phylogenetic assignment of each gene in the original gene catalog and summed the relative abundance of genes under a certain taxonomic rank to obtain the abundance of each taxonomic rank. Finally, all the abundance information was obtained from phylum, genus and species levels to establish the taxonomic abundance profile. The functional abundance profile was constructed by the same procedure.

### 2.5. Similarity Analysis of Bacteria Species

The correlation of various microbial communities was analyzed by the Jaccard similarity coefficient and Bray–Curtis similarity matrix to compare their similarities and differences [28,29]. The former only considers the presence or absence of microbial species when calculating the correlation, but the latter considers not only the presence of microbial species, but also the relative abundance of species. Both methods were calculated by the vegdist function in vegan package in R software (version 3.6.2, Murry Hill, NJ, USA).

### 2.6. Statistical Analysis

All statistical analysis was performed in R software (version 3.6.2, Murry Hill, NJ, USA). The Wilcoxon rank sum test (*p* < 0.05) and LOGD value (LOGD > 1) were used to test the differential abundance of microorganisms and functional orthologues. *P* values were adjusted to control the False Discovery Rate (FDR) [30]. LOGD is a normalized value to calculate the difference abundance between two groups of samples using the following formula:

(1) $$\mathrm{LOGD}={log}_{10}\left(abundance\;of\;disease\;group-abundance\;of\;healthy\;group\right)$$

## 3. Results

### 3.1. Basic Workflow and Characteristics of Three Different Metagenomics Profiling

**Figure 1:**
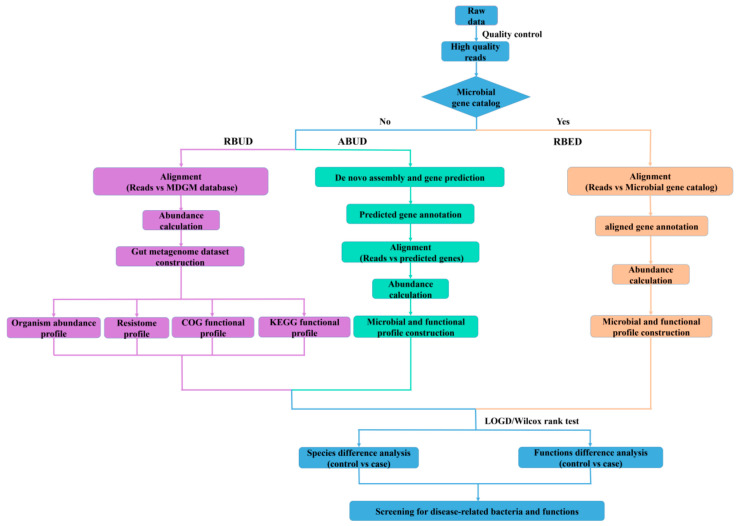
Flow chart of the read-based metagenomics profiling for unestablished database (RBUD) method, the assembly-based metagenomics profiling for unestablished database (ABUD) method and the read-based metagenomics profiling for established database (RBED) method to analyze metagenomic data in this study. The blue box represents the common steps of all methods. The purple box represents the steps of RBUD method. The light green box represents the steps of ABUD method, and the orange box represents the steps of RBED method.

In this study, we employed two conventional methods (assembly-based metagenomics profiling for unestablished database (ABUD) and read-based metagenomics profiling for established database (RBED)) and our newly developed method (RBUD) to analyze the intestinal microbial metagenomes of avian colibacillosis chicken and T2D patients, comparing the accuracy and effectiveness of the three methods [8]. The flow chart showed the difference of the three analyses (Figure 1). These three methods can be distinguished by whether there is an established reference gene catalog and whether the contigs are assembled.

#### 3.1.1. Read-Based Metagenomics Profiling for Unestablished Database (RBUD)

RBUD was developed in this study to analyze metagenome data without assembly steps. The first important step for RBUD was to establish a relevant database, especially for the rare types of samples. In this study, we built a metagenome database (MDGM) based on the data of microorganisms from National Center for Biotechnology Information (NCBI) [17]. It contains microbial species from different hosts, different environmental sources and different sampling parts of the same host, which are competent for most metagenomic studies. To fulfill the construction of MDGM, there were several sub-steps that needed to be done as follows: firstly, whole microbial genome data (5133 bacteria, 9548 viruses and 243 fungi) and corresponding species and their taxonomic annotation information were downloaded from the NCBI database to construct the microbial species dataset (on 3 December 2015) [17]. Secondly, the CDS(coding sequence) region sequences and corresponding annotations of these sequences including gene ID, protein ID, location in chromosome, Cluster of Orthologous Group of Proteins (COG) function and protein product were collected to build the functional dataset. Most of the functional annotations can be obtained according to gene ID or protein ID of the NCBI database [17]. When some databases do not support information retrieval by NCBI gene ID or protein ID, the sequence of the CDS region was aligned with the nucleic acid or protein sequence in these databases through BLASTN or BLASTP search (e-value < 1 × 10^−5^) to obtain functional annotation [26]. The databases that we have used for functional annotation in this study were eggNOG [31], Kyoto Encyclopedia of Genes and Genomes (KEGG) [32], Antibiotic Resistance Gene Database (ARDB) [33], Carbohydrate-Active enZymes Database (CAZy) [34], The Comprehensive Antibiotic Resistance Database (CARD) [35], Universal Protein (UniProt) [36] and Metabolic Pathways From all Domains of Life (MetaCyc) [37].

The second step of RBUD was to single out the high-quality reads. In this step, the raw data were obtained by high-throughput sequencing or from published data. Then, quality control was carried out to remove low-quality reads and host DNA contamination.

The third step of RBUD was to establish microbial species profiling and functional profiling of our testing samples. In this step, we firstly aligned the high-quality reads with MDGM to calculate the abundance of all microbiome species. Then, we started to analyze the difference of microbial composition among sample groups and to calculate the microbial diversity. Meanwhile, the high-quality reads were also aligned with the CDS region sequences of MDGM to calculate the abundance of all genes and obtain their functional annotations. The genes were clustered by their functions to get multiple functional orthologues containing different genes. Then, redundant genes with the same abundance and in the same functional orthologue were removed. The functional abundance was the sum of the non-redundant gene abundance in the same functional orthologue. Subsequently, the differential genes were identified, and functional analyses were performed.

The codes used in the RBUD method and for constructing MDGM database have been successfully uploaded to https://github.com/DMsiast/RBUD.git, which is open to all users. Researchers can download the full processing code from the website. For the purpose of studying bacteria, viruses and fungi separately, we provided three individual databases that can save time and improve accuracy.

#### 3.1.2. Assembly-Based Metagenomics Profiling for Unestablished Database (ABUD)

ABUD is a kind of commonly used method to analyze microbial shotgun genome data, which can be applied without an existing reference database [8]. RAST [38], Megan4 [39], MOCat2 [40], Carnelian [41] and IMG4 [42] belong to ABUD method. The basic principal and the main workflows of these tools are similar. First, low quality reads and host DNAs are removed from raw data. Then, the ORFs are obtained after contigs assembly and gene prediction by MetaGeneMark software. The predicted genes are annotated via aligning ORFs with a universal database (e.g., MDGM), and a non-redundant reference gene catalogue is built. After that, high quality sequencing reads are aligned with the above gene catalogue to calculate gene abundance capable of building microbial and functional profiles. Finally, the characteristics of microflora can be acquired through the comparative analysis of different sample groups. However, for the ABUD method, the utilization rate of sequencing reads is reduced during data processing and the loss of biological information is serious. Although increasing sample size and sequencing depth can solve this problem in a certain degree, more computing resources and economic investments are required.

#### 3.1.3. Read-Based Metagenomics Profiling for Established Database (RBED)

The RBED method can be implemented through external sequence data sources (such as open reference genomes) without reads assembly [8]. Since assembly is a slow, resource intensive and lossy process, reads directly mapping to the existing database is the core concept for RBED. MG-RAST [43], ShotMap [44], COGNIGER [45] and HUMAnN2 [46] belong to RBED method, which have similar procedures with different reference databases. For the RBED method, the data pretreatment of RBED is consistent with that of ABUD. Then, the high-quality reads are aligned with the reference gene catalog, which has been built in the existing database to calculate the relative abundance of these genes. Retrieving the gene annotation information in the gene catalog, the bacterial species and functional profiles are established. Finally, through the comparative analysis of different sample groups, the microflora characteristics can be obtained.

The RBED approach can mitigate the assembly problems, speed up computation and analyze the low abundance microorganisms that cannot be assembled. Nowadays, many reference genomes are rapidly increasing [47,48], and each reference genome can be used for the analysis of a certain sample type, such as the human gut [49]. However, the lack of representative reference genome hampers the analysis of some more diverse environments, such as soil and oceans. Thus, the application scope of the RBED method based on the existing reference database is limited by the source of samples.

### 3.2. Comparisons of Three Different Metagenomics Profiling in a Case-Control Study of Type 2 Diabetes Mellitus

The incidence of obesity and T2D is surging in the world and the intestinal metagenomic data in T2D patients are accumulating fast [50]. To illustrate the effectiveness of the RBUD method, metagenomic data of 10 healthy controls and 10 T2D cases were analyzed by the three methods, evaluating the advantages and disadvantages. Using whole microbial genome sequencing, the averages of 2.95 Gb of high-quality data were obtained, which were used to analyze the alterations in species and functions between T2D and control samples. The procedure of the ABUD method to analyze metagenome data in this case was consistent with what we have described above. In order to show only the effect of whether reads are assembled or not on the results of RBUD and ABUD, MDGM was adopted as reference database in the two methods. To study the intestinal microbes in T2D patients, a taxonomic abundance profile and a functional abundance profile were established based on MDGM. The taxonomic abundance profiles were established with three levels, phylum, genus and species. The functional abundance profile included Cluster of Orthologous Group of Proteins (COG) sub-functional profile and KEGG sub-functional profile. Additionally, the procedure of the RBED method to analyze metagenome data was consistent with what we have described above. The Metagenomics of the Human Intestinal Tract (MetaHIT) was a representative metagenome database using in RBED analysis.

#### 3.2.1. RBUD and ABUD Exhibited Similarity in Identifying Characteristics of Intestinal Microbiota, Rather than RBED in T2D Cases

**Figure 2:**
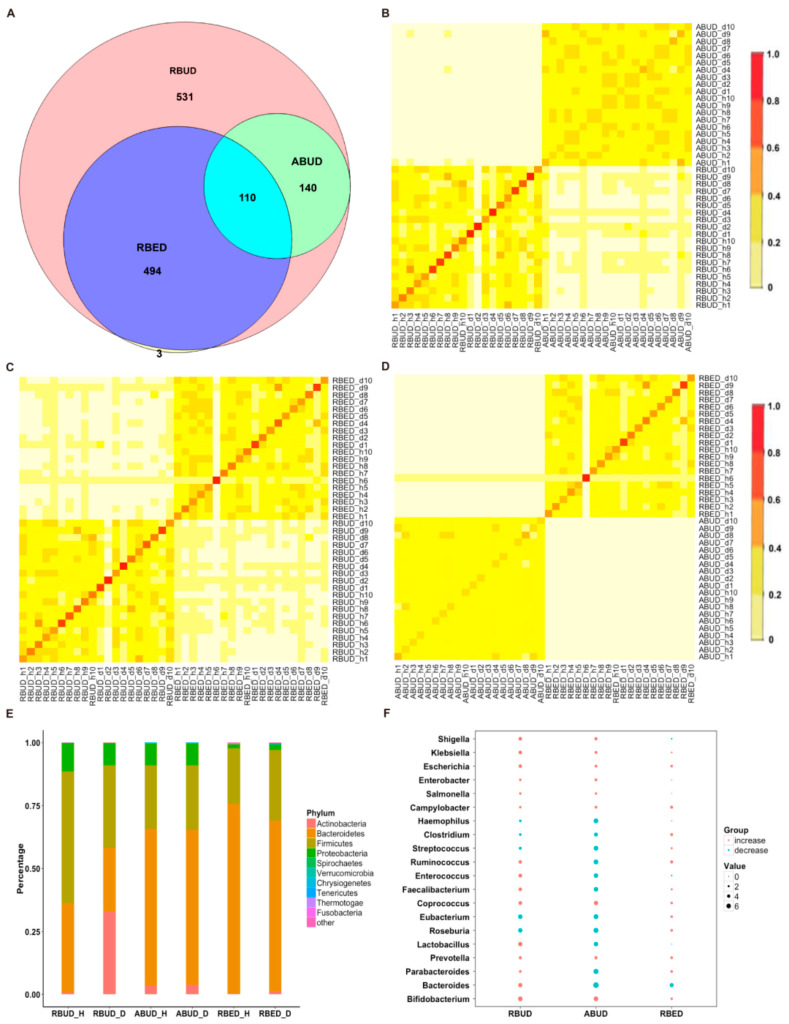
The comparison of intestinal microbial changes between diseased and healthy samples which were analyzed by the three methods in the case study of T2D. (**A**) Venn Diagram showed the specific and the common bacterial species identified by RBUD, ABUD and RBED methods. (**B**) The species similarity between the RBUD and ABUD method using Bray–Curtis similarity analysis, in which the shades of color represent the degree of correlation. The first and third quadrants represent the similarity of bacteria species in all samples using the same method. The second and fourth quadrants represent the similarity of bacteria species in all samples using different methods. (**C**) The species similarity between RBUD and RBED using Bray–Curtis similarity analysis. (**D**) The species similarity between ABUD and RBED using Bray–Curtis similarity analysis. (**E**) The changes of the abundance percentage of gut microbiota between healthy and T2D samples at the phylum level. (**F**) The changes of abundance of genera that obtained by different methods.

By comparing the microbial species obtained using the three methods, it was found that the microbes identified by the RBUD method could cover all the microbes acquired by the other two methods (Figure 2A). The correlation analysis of microbial composition obtained by ABUD, RBUD and RBED was carried out pairwise by the Jaccard similarity coefficient and Bray–Curtis similarity matrix. The correlations of microbial composition were shown in Figure 2B–D and Figure S1A–C. There was obviously a correlation between RBUD vs. ABUD, and RBUD vs. RBED, but no correlation was found between ABUD and RBED. These results suggested that the RBUD method was more comprehensive than other methods in species analysis.

These three methods determined the dominant bacteria at the phylum level, including *Bacteroidetes*, *Firmicutes*, *Proteobacteria* and *Actinobacteria*. However, the RBUD method was more effective in comparing the changes of microflora composition between the disease group and healthy group with an obvious alteration (Figure 2E, Figure S2A). The significant differential species between disease and healthy groups were identified by RBUD, ABUD and RBED. The genera containing these differential species were used to compare the result similarity of the three methods. It showed that RBUD and ABUD were more consistent, but RBED was different from the others (Figure 2F). Due to the limitation of sample source in the MetaHIT database, the results of the RBED method were different from those of the other two methods in both intestinal flora composition and microbial variation.

To further evaluate the effectiveness of different methods in species identification, the intestinal flora characteristics obtained by the three approaches were compared with the documented experimental studies in T2D. In the analysis of RBUD and ABUD, both found that the number of butyric acid producing strains of *Clostridium* and *Roseburia* were reduced and the *Lactobacillus* and *Escherichia* that attached to *Proteobacteria* were increased in T2D patients, which was consistent with the existing research [16,51,52,53]. However, the RBED method did not find the same trends, which was not in agreement with previous reports (Figure 2F).

In summary, the three methods have different performances for the species identification. The RBUD method and the ABUD method have similar ability in finding intestinal microbes, but the RBED method is not as good as them. Since the MDGM database was used to annotate the species in RBUD and ABUD methods, while the MetaHIT database [11] was adopted to annotate the species in RBED method, it suggests that the MDGM database has more advantages than other metagenome databases in species analysis. The only different key step between RBUD and ABUD is assembly or not, while RBUD has advantages in saving time and computing resources.

#### 3.2.2. RBUD and RBED Detecting Similar Microbial Functions and More Disease-Related Functions than ABUD

**Figure 3:**
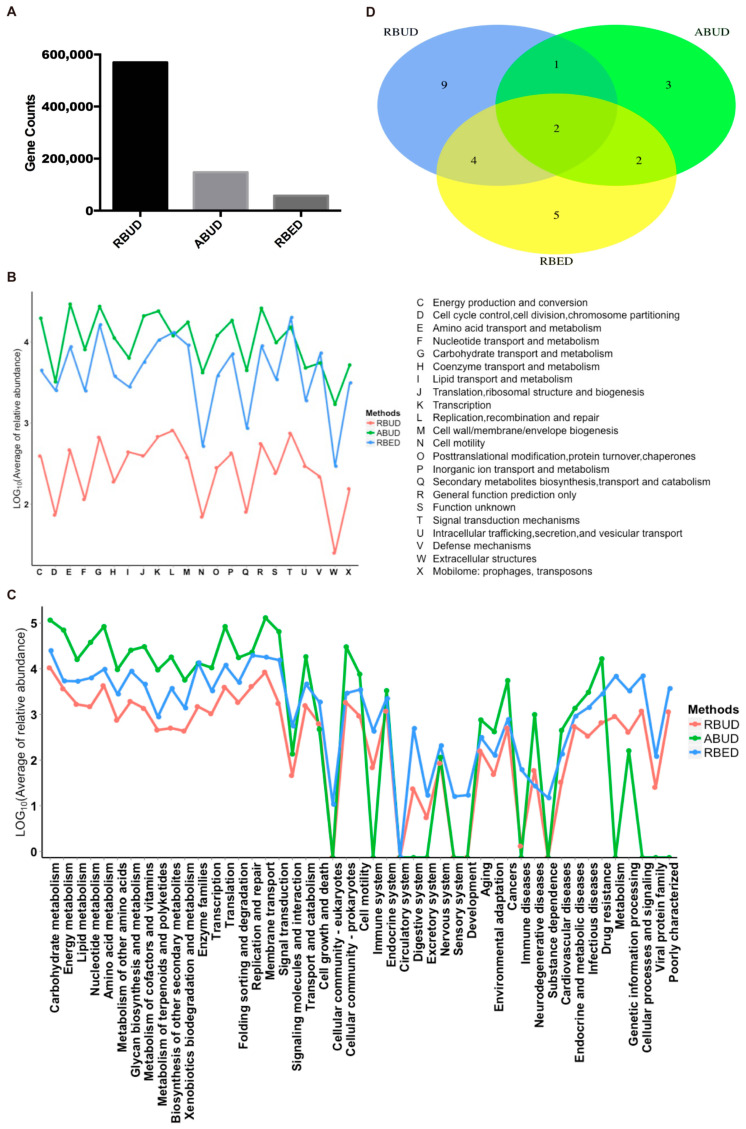
The comparative analysis of the enriched functions using the three methods in the case study of T2D. (**A**) The comparison of enriched gene counts using the three methods. (**B**) The relative abundance trend of functions in categories of Cluster of Orthologous Group of Proteins (COG) using different methods. (**C**) The relative abundance trend of functions in categories of Kyoto Encyclopedia of Genes and Genomes (KEGG) using different methods. (**D**) Venn diagram showed the overlap of KEGG functional modules that enriched by different methods.

For the gene and function annotation, we observed that RBUD computed more genes than the other two methods (Figure 3A). In the functional enrichment analysis of COG and KEGG, the distribution trend of RBUD and RBED results was similar in terms of functional categories, and it was obviously different from that of ABUD (Figure 3B,C). In addition, the differential KEGG functions between T2D and healthy patients found by the RBUD and RBED methods had more overlap. Among these overlap functions, the cofactor and vitamin biosynthesis, aromatic amino acid metabolism, bacteria secretion system, fatty acid metabolism and other carbohydrate metabolism have been reported to be associated with T2D (Figure 3D, Table 1) [54,55,56,57,58]. However, the functional enrichment by the ABUD method had no T2D related functions (Table 1). During the process of constructing a non-redundant gene dataset in the ABUD method, the contigs assembly, ORF prediction and annotation of species and functions of predicted genes will lose a lot of biological information, which makes the ABUD method find no T2D-related function in the gut microflora.

To sum up, RBUD enriched more disease-related genes and functions than ABUD, which was more consistent with RBED. The existing metagenome database adopted by RBED usually contain a large number of samples, which makes RBED more comprehensive for functional annotations. For ABUD, information loss affected the accuracy of functional analysis, especially in the study of a small sample size. Therefore, RBUD and RBED are similar in function enrichment analysis, indicating that RBUD is competent for the study of small samples.

### 3.3. Comparisons of Three Different Metagenomics Profiling in A Case-Control Study of Avian Colibacillosis

Compared with human and mouse, the intestinal microflora of chicken are less studied. Because of the obvious characteristics of intestinal flora and clear pathogenic mechanism of avian colibacillosis, we generated gut metagenomic data in nine avian colibacillosis and nine control chicken samples, and analyzed them by RBUD, ABUD and RBED methods, respectively, to evaluate the advantages and disadvantages of the three methods. The whole metagenome shotgun sequencing was performed to obtain an average of 3.04 Gb high quality data. The procedures of ABUD and RBED methods to analyze metagenome data in this case were consistent with what we have described above. The MDGM was adopted as the reference database for ABUD and RBUD. The taxonomic abundance profiles were established with three levels: phylum, genus and species. For this study, the functional abundance profile included an additional antibiotic resistome sub-functional profile except for COG and KEGG sub-functional profiles. For the RBED method, the Chicken Gut Microbial Gene Catalog [12] was a representative metagenome database using RBED analysis.

#### 3.3.1. Three Metagenome Methods Exhibited Similarity in Identifying the Characteristics of Enterobacteria in the Avian Colibacillosis Individuals

**Figure 4:**
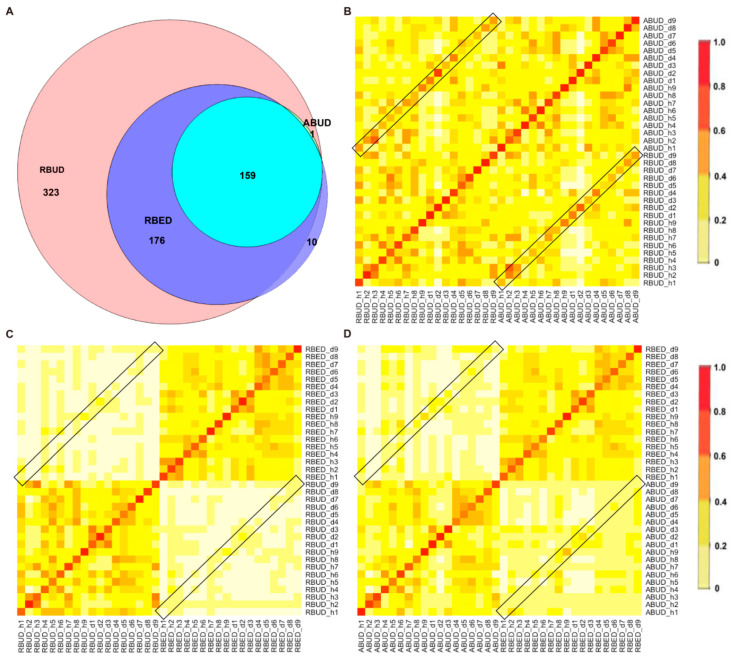
Changes of the microbial community detected by the three methods between healthy group and avian colibacillosis group. (**A**) Venn Diagram showed the specific and the common bacterial species identified by RBUD, ABUD and RBED. (**B**) Species similarity between RBUD and ABUD using Bray–Curtis similarity matrix correlation analysis. The box represents the similarity of bacteria species in the same sample using different methods. (**C**) Species similarity between RBUD and RBED using Bray–Curtis similarity matrix correlation analysis. (**D**) Species similarity between ABUD and RBED using Bray–Curtis similarity matrix correlation analysis.

By comparing the microbial species obtained by the three methods, it was found that the microbes identified by the RBUD method could include most of the microbes acquired by the other two methods (Figure 4A). Unsurprisingly, the microbial composition obtained by the RBUD and ABUD methods was correlated in each sample, which was shown in the boxes of a Bray–Curtis correlation analysis chart (Figure 4B). However, the correlation of microbial composition between RBED and the other two methods was very weak (Figure 4C,D). The Jaccard similarity coefficient analysis which does not consider the abundance of species showed no significant pairwise correlation of the three methods. This illustrated that the abundance of microbial species was of great significance in analyzing the similarity of microflora (Figure S1D–F).

**Figure 5:**
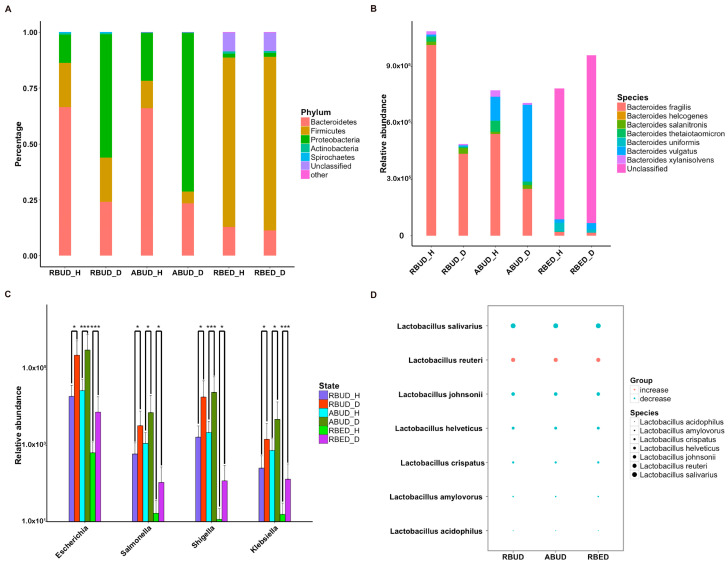
Characteristics of gut microbiota at different taxonomic levels using these three methods in avian colibacillosis. (**A**) The changes of the abundance percentage of gut microbiota between healthy and diseased cases at the phylum level. (**B**) The abundance change of species that belongs to the genus of Bacteroides. (**C**) The significant abundance change of the dominant genus that belongs to Proteobacteria. * adjusted *p* < 0.05; *** adjusted *p* < 0.01. (**D**) The trend changes of species abundance between healthy and disease groups detected by the three methods.

Subsequently, the composition of gut microbes was analyzed in detail from phylum, genus and species. All three methods found that *Bacteroidetes*, *Firmicutes* and *Proteobacteria* were the dominant phyla in all samples, and *Proteobacteria* increased dramatically in disease samples. However, from the results of RBUD and ABUD, the microbial composition showed a significant difference between the healthy group and disease group, but it was not significantly different in the result of RBED (Figure 5A, Figure S2B). At the genus and species level, the relative abundance of *Bacteroides fragilis*, which belongs to *Bacteroides*, in the disease group was significantly lower than that in healthy group, accounting for 97% of total number of *Bateroidete* (Figure 5B, Figure S3A). In contrast, the abundance of *Escherichia*, *Salmonella*, *Shigella* and *klebsiella*, which all belong to *Proteobacteria*, increased significantly in the disease group (Figure 5C). In addition, the change trend of *Lactobacillus* was similar (Figure 5D, Figure S3B). These findings were consistent in the three methods.

**Figure 6:**
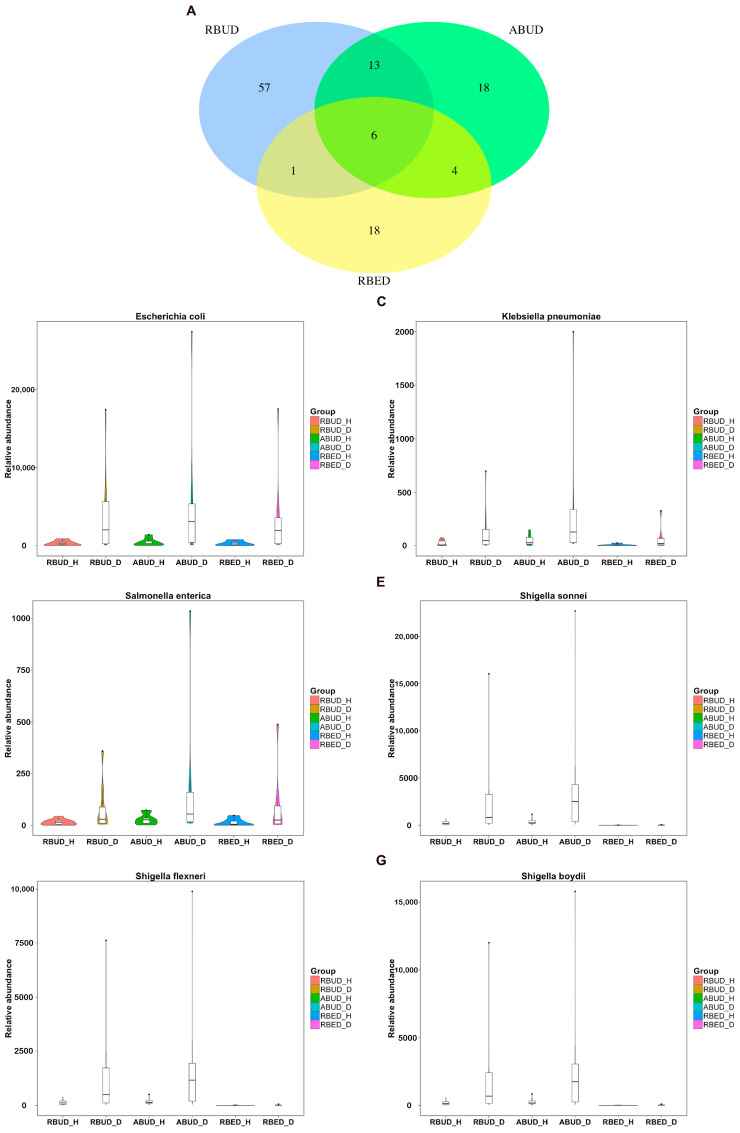
The relative abundance changes of the common gut microbiota detected by the three methods. (**A**) Venn Diagram showed the specific or the common differential microbial species obtained by the three methods in healthy and disease samples. (**B**) The abundance change of *Escherichia coli* between healthy and disease groups. (**C**) The abundance change of *Klebsiella pneumonia*. (**D**) The abundance change of *Salmonella enterica*. (**E**) The abundance change of *Shigella sonnei*. (**F**) The abundance change of *Shigella flexneri*. (**G**) The abundance change of *Shigella boydii*.

By analyzing the different strains between the disease and the healthy groups obtained by each method, we found that RBUD and ABUD obtained more overlapped strains. Combining the results of three methods, there were six strains of bacteria overlapped, which were all related to avian colibacillosis, including *Escherichia coli*, *Klebsiella pneumonia*, *Salmonella enterica*, *Shigella boydii*, *Shigella flexneri* and *Shigella sonnei* (Figure 6A–G).

Since the chicken reference database used in the RBED method contains a large sample size, in the chicken case study, the three methods showed similarity in the detection of microbial components. This proves again that RBUD is an effective method for species analysis, especially considering the time and computational cost.

#### 3.3.2. RBUD and RBED Detecting Consistent Microbial Functions and More Disease-Related Functions than ABUD

**Figure 7:**
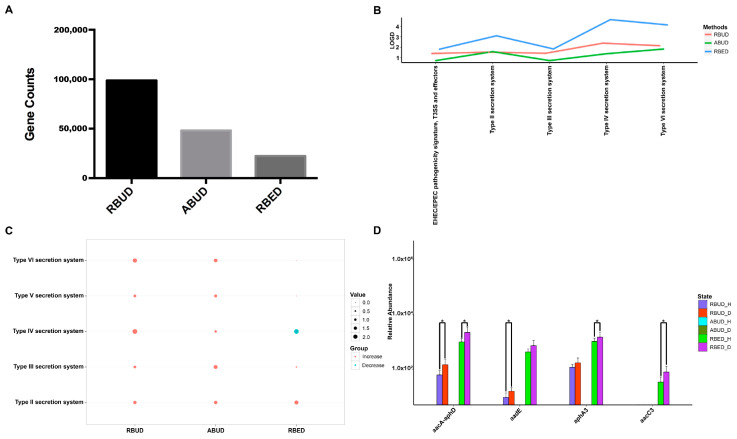
Enriched functions in disease samples identified by the three methods in the study case of avian colibacillosis. (**A**) The counts of the enriched genes obtained by the three methods. (**B**) The abundance changes of the pathogenic functions of bacterial secretion system in the COG database. (**C**) The abundance changes of the functional modules of bacterial secretion system in the KEGG database. (**D**) The significant change of antibiotic resistance gene expression detected by the three methods between the healthy and disease groups. _H represents the healthy group; _D represents the disease group. *adjusted *p* < 0.05.

It is well known that pathogenic *Escherichia coli* is the main pathogenic bacteria, and its adhesion and virulence factors are secreted by Type I-VI secretion system (T1SS-T6SS) [59]. Therefore, the number of enrichment genes, bacteria secretion system and antibiotic resistance genes were conducive to the evaluation of the three methods. Notably, the number of enriched genes in the analysis result of RBUD was twice than that in ABUD and four times than that in RBED (Figure 7A). In COG functional enrichment analysis, almost all functions of bacteria secretion system were increased in disease group, which was detected by all three methods (Figure 7B). Moreover, KEGG functional analysis showed that RBUD and RBED were better than ABUD in functional enrichment of bacteria secretion system in disease samples (Figure 7C). The RBUD and RBED enriched aminoglycoside antibiotic resistance genes in disease group, which was consistent with the fact that the chickens were fed with antibiotics. However, the ABUD method did not detect any changes in antibiotic resistance genes (Figure 7D). The RBUD method can steadily reflect the disease-related functions in an avian colibacillosis case study, which is consistent with its performance in the T2D case study.

## 4. Discussion

Currently, a large number of metagenome databases have been established for better analyze the metagenomic data [8]. In order to improve the coverage of microbial diversity, extensive samples are required to construct metagenome database [60]. The databases were usually constructed based on the data derived from several relevant studies. However, since it is hard to include all disease types, sample sources and host species in several specific studies, the limitations of the established databases still exist. The MDGM contains a variety of microorganism from different sources, which could overcome the limitations of existing databases. In addition, by extracting biological information from a reference-based and assembly-based database, the MDGM database will be constantly updated and improved. This will help us to analyze the genomes of special or newly discovered organisms.

To evaluate the performance of RBUD, we compared the results of metagenomics data analysis with the other two kinds of commonly used methods. Through the metagenomic data analysis in T2D and avian colibacillosis, we found that the results of RBUD method and ABUD method were consistent in species analysis, and the RBUD method had more advantages than RBED in targeting key strains of the disease. RBED needs a well-established metagenomic database. Although the database contains a large sample size, its sample source is often relatively single. That is why RBED cannot identify as many species as RBUD and ABUD. Nevertheless, the RBED method has detected the disease-related bacteria in an avian colibacillosis study, which was not found in the T2D study. There are three possible reasons for this result. One reason is that the complexity of T2D pathology leads to the heterogeneity of bacterial composition among individuals. However, avian colibacillosis has relatively clear pathogenic bacteria, and the diversity of bacterial composition is lower among individuals. Another reason is that the chicken gut microbial gene catalog includes chicken samples from the Rose 308, but the MetaHIT database does not contain any data of Chinese diabetes patients. The third reason is that in the known metagenome databases, the annotation rate of most genes at the species level is only 30%, which may cause the deviation of relative abundance of gut microbiota. These results illustrated that the existing known metagenome databases is limited by the source of samples, resulting in incomplete information, which directly affects the subsequent analysis.

For the functional analysis of the microbial community, if there is no established metagenomic database, it is necessary to establish a specific database, which is based on reads assembling and ORF predicting. The samples in that database only include the samples sequenced in that study. The relatively small sample size will lead to incomplete functional information of the database, and a large amount of information will be lost during the data processing, which will seriously affect the accuracy of functional analysis. In contrast, the reference database, which the RBED method relies on, has the advantages of a large sample size, which can display the microflora function more comprehensively and systematically. In fact, by comparing the results from the three methods, we found that RBUD and RBED could enrich the same functional characteristics and identify more disease related functions than ABUD. Therefore, all the results show that the RBUD method cannot only achieve similar performance as RBED method in functional annotation, but also be competent for small samples. In addition, the results from RBUD method were consistent in T2D and avian colibacillosis studies, which also proved the universality and effectiveness of the method.

In conclusion, a simple and rapid functional analysis method (RBUD) was developed in this study, which makes full use of sequencing reads and improves the utilization of data. The RBUD method combined with the MDGM database has great advantages in species and functional analysis, especially for small sample size studies without a metagenome database. At the same time, RBUD greatly improves the speed of data analysis and reduces the cost of establishing a microbial gene catalog based on a large number of samples. We believe it will provide a great convenience for further metagenomic study.

## Figures and Tables

**Table 1:** The enriched common function characteristics of RBUD, ABUD and RBED.

Functions	RBUD	ABUD	RBED
Ribosome	↑	↑	↑
Cofactor and Vitamin biosynthesis	↑	↓	↑
Aromatic amino acid metabolism	↑	-	↑
Bacterial secretion system	↑	-	↓
Fatty acid metabolism	↑	-	↑
Other carbohydrate metabolism	↑	-	↑
Carbon fixation	-	↓	↑
Mineral and organic iron transport system	-	↑	↑
Two-component regulatory system	↑	↑	-

Note: ↑ represents the relative abundance of the function was increased in T2D; ↓ represents the relative abundance of the function was decreased in T2D; - represents the relative abundance of the function was not enriched.

## Supplementary Materials

The following are available online at https://www.mdpi.com/2076-2607/8/10/1563/s1, Figure S1: Comparison of the similarity between the two in three methods using Jaccard Coefficient similarity matrix correlation analysis in avian colibacillosis and T2D; Figure S2: Relative abundance of log10 at the phylum level in T2D and avian colibacillosis; Figure S3: The abundance alterations of Bacteroidetes and Firmicutes in avian colibacillosis; Table S1: Phenotype information of all chicken samples in both healthy and disease groups; Table S2: Basic Information of sequencing data of 18 broiler chickens; Table S3: Bioassay and clinical information for the 20 people in the cohort; Table S4: Basic Information of sequencing data of 20 people.
