# Supplementary material for: RBUD: A New Functional Potential Analysis Approach for Whole Microbial Genome Shotgun Sequencing
Source: Microorganisms. 2020 Oct 10;8(10):1563. doi: 10.3390/microorganisms8101563 (PMC7650719; doi:10.3390/microorganisms8101563)
Supplement: Supplementary file 1 [file microorganisms-08-01563-s001.zip › microorganisms-951933-supplementary-original/microorganisms-951933-supplementary figures-original.pdf]

Supplementary Figures

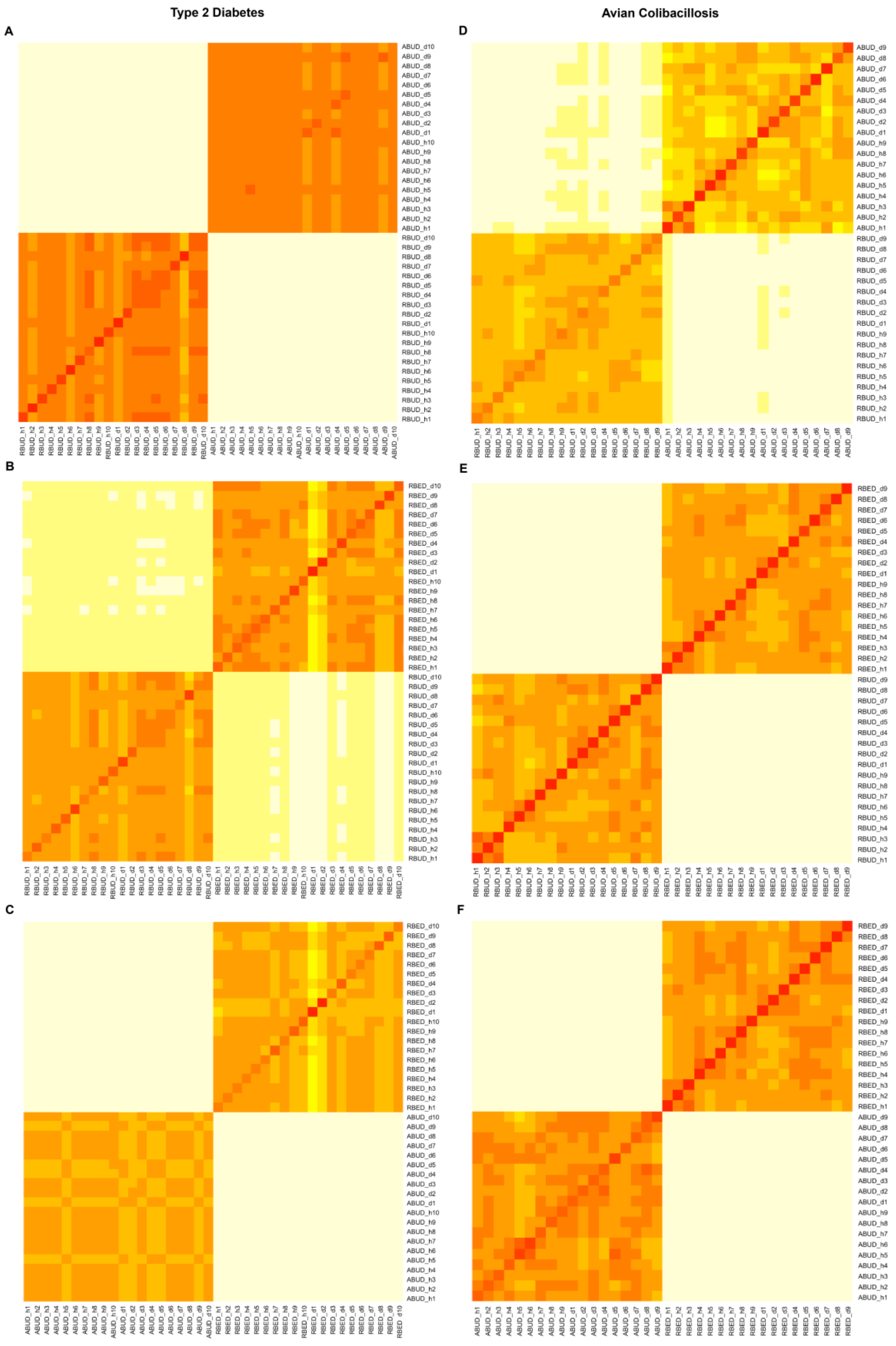

Supplementary Figure 1. Comparison of the similarity between the two in three methods using Jaccard Coefficient similarity matrix correlation analysis in avian colibacillosis and T2D. **(A)** Species similarity between RBUD method and ABUD method in T2D. **(B)** Species similarity between RBUD method and RBED method in T2D. **(C)** Species similarity between ABUD method and RBED method in T2D. **(D)** Species similarity between RBUD method and ABUD method in avian colibacillosis. **(E)** Species similarity between RBUD method and RBED method in avian colibacillosis. **(F)** Species similarity between ABUD method and RBED method in avian colibacillosis.

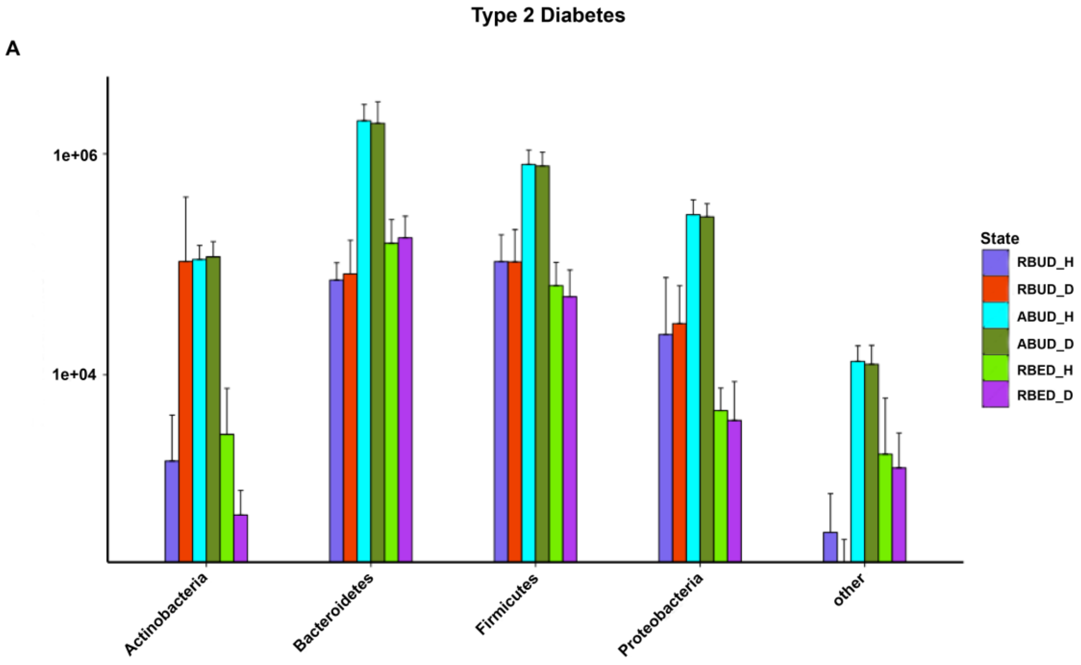

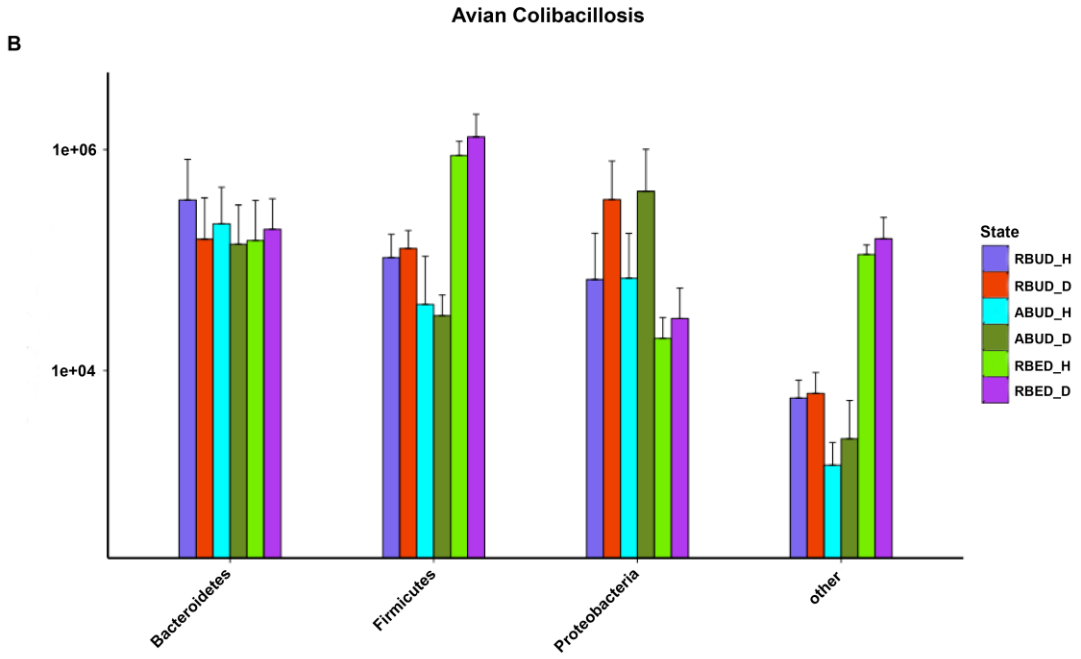

Supplementary Figure 2. Relative abundance of log10 at the phylum level in T2D and avian colibacillosis. **(A)** The alterations of relative abundance for dominant phylum between healthy controls and disease cases in T2D. **(B)** The abundance change of dominant phylum between healthy group and disease group in avian colibacillosis.

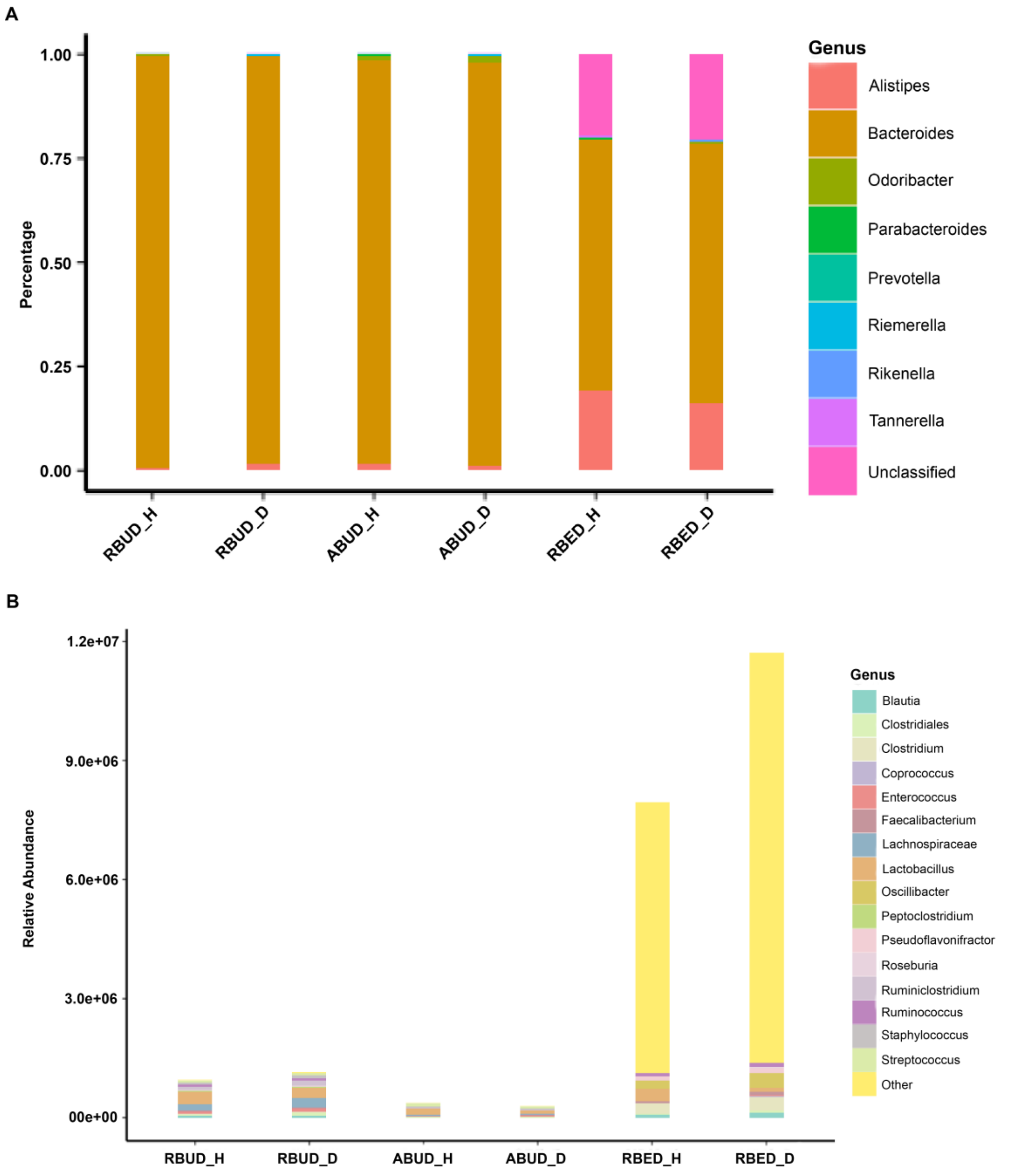

Supplementary Figure 3. The abundance alterations of Bacteroidetes and Firmicutes in avian colibacillosis. **(A)** The percentage change of Bacteroidetes. **(B)** The relative abundance alteration of Firmicutes.
